# Intranasal Administration of Agomir-let-7i Improves Cognitive Function in Mice with Traumatic Brain Injury

**DOI:** 10.3390/cells11081348

**Published:** 2022-04-15

**Authors:** Xuan-Cheng He, Jian Wang, Hong-Zhen Du, Chang-Mei Liu, Zhao-Qian Teng

**Affiliations:** 1 State Key Laboratory of Stem Cell and Reproductive Biology, Institute of Zoology, Chinese Academy of Sciences, Beijing 100101, China; hexuancheng@ioz.ac.cn (X.-C.H.); wj980411@163.com (J.W.); duhongzhen@ioz.ac.cn (H.-Z.D.); 2 Institute for Stem Cell and Regeneration, Chinese Academy of Sciences, Beijing 100101, China; 3 Beijing Institute for Stem Cell and Regenerative Medicine, Beijing 100101, China; 4 Savaid Medical School, University of Chinese Academy of Sciences, Beijing 100049, China

**Keywords:** traumatic brain injury, let-7i, learning and memory, agomir, *STING*

## Abstract

Overcoming the lack of drugs for the treatment of traumatic brain injury (TBI) has long been a major challenge for the pharmaceutical industry. MiRNAs have emerged as potential targets for progress assessment and intervention against TBI. The brain-enriched miRNA let-7i has been proposed as an ideal candidate biomarker for TBI, but its regulatory roles in brain injury remain largely unknown. Here, we find that the expression of let-7i is significantly downregulated in the early stages of a hippocampal stab wound injury. The noninvasive intranasal administration of let-7i agomir significantly improves cognitive function and suppresses neuroinflammation, glial scar formation, and neuronal apoptosis in TBI mice. Mechanically, *STING* is a direct downstream target of let-7i after brain injury. Furthermore, the intranasal delivery of let-7i agomir can also effectively inhibit *STING* and is beneficial for inflammation resolution and neuronal survival in a mouse model of pial vessel disruption stroke. Consequently, let-7i agomir is a promising candidate for clinical application as a chemically engineered oligonucleotides-based therapeutic for brain injury.

## 1. Introduction

Traumatic brain injury (TBI) is one of the leading causes of death and permanent disability worldwide and is a risk factor for developing dementia and neuropsychological diseases [1]. Although much is known concerning the mechanisms of TBI, including excitotoxicity, acidotoxicity, oxidative stress, inflammation, ionic imbalance, and apoptosis [2], there is no efficacious treatment for patients following TBI [3]. TBI can induce severe physical, emotional, and cognitive dysfunctions [4,5], and early intervention is believed to be the key to minimizing the pathophysiology that aggravates the symptoms [6,7].

MicroRNAs (miRNAs) are small single-stranded non-coding RNA molecules (about 22 nucleotides) that govern the processes of RNA silencing and the post-transcriptional regulation of gene expression. As a single miRNA regulates the expression of multiple target genes involved in many cellular processes, manipulating the expression of a single miRNA may affect the entire gene network, thus changing the phenotype of complex diseases. Mounting evidence suggests that miRNAs may serve as potential targets for progress assessment and intervention against TBI because they control a range of physiological and pathological functions, such as cell proliferation, differentiation, apoptosis, and metabolism [8,9].

Let-7 is a family of evolutionary conserved miRNA that are highly expressed in adult tissues and generally serve as key regulators of developmental processes [10]. Let-7i has been considered as an ideal candidate biomarker for TBI due to its elevated levels in both serum and cerebrospinal fluid immediately after blast wave exposure [11]. In contrast, the level of let-7c-5p is rapidly reduced in TBI brains and gradually recovers to a normal level 14 days after TBI [12]. The intracerebroventricular injection of let-7c-5p attenuates TBI-induced neurological dysfunction and brain edema in mice, suggesting its therapeutic potential for improving the neurological outcome of TBI [12]. Furthermore, several studies demonstrate that let-7 miRNAs are important modulators of neuroinflammatory processes by targeting the CCAAT/enhancer-binding protein-δ (C/EBP-δ) [13], the Toll-like receptor 4 (TLR4) [14], as well as the cytokines IL-6 and IL-10 [15]. Together, these findings indicate that let-7 may be a crucial therapeutic target for neurotrauma.

Here, we aimed to examine whether TBI affects let-7 expression in the brain and to assess whether the restoration of let-7 levels is beneficial for suppressing neuroinflammation and reducing neuronal apoptosis after TBI. Furthermore, the mechanism of let-7 targeting for TBI was also explored.

## 2. Materials and Methods

### 2.1. Animals

C57BL/6J mice were obtained from the SPF (Beijing, China) Biotechnology company and maintained in groups of 3~5 animals on a 12 h light/dark cycle, and they had free access to water and a standard mouse diet. Two-month-old male mice were assigned to undergo TBI or sham surgery. All animal procedures followed the ethical guidelines for the care and use of experimental animals, and all experiments were approved by the Animal Committee of the Institute of Zoology, Chinese Academy of Sciences.

### 2.2. Hippocampal Stab Injury

Hippocampal stab injury (HSI) was performed as previously described with minor modifications [7]. Two-month-old male mice were anesthetized by intraperitoneal injection of Avertin (200 mg/kg body weight) and received a hippocampal stab wound injury in a KOPF stereotaxic apparatus. A thin blade was used to open a cranial window (3 mm in diameter) on the parietal skull, and a sterile surgical scalpel blade (JINHUAN, Yancheng, China; #11) was inserted in the hippocampus by positioning it parallel to the brain surface at 2.5 mm posterior to bregma, 2.3 mm lateral to the sagittal suture, and 3 mm below the dura. Finally, the craniotomy was sealed with bone wax (SANYOU, Shanghai, China).

### 2.3. Pial Vessel Disruption (PVD) Stroke

PVD strokes were induced as previously described [16]. Briefly, mice were anesthetized, and the skull and dura were removed in the region bound by 0.5 to +2.5 mm anterior/posterior and +0.5 to 3 mm midline/lateral, relative to bregma. The medium-sized pial vessels in the exposed region were removed using a saline-soaked cotton swab.

### 2.4. Intranasal Delivery of Agomir-let-7i

Intranasal administration of agomir-let-7i was conducted as described previously [17]. Agomir-let-7i (RIBO Biotech, Guangzhou, China) was dissolved in 24 µL of RNase-free water (1 nmol) and administered by pipette in 4-µL drops (total of six fractions), alternating between each nostril every 2–3 min. Control mice received an equal volume of scramble nucleotides, which have no biological function but the same molecular weight as agomir-let-7i. Agomir-let-7i or scramble was delivered to the brain every other day for a total of 14 days. Then, the mice were trained and tested on the rotarod and the Barnes maze.

### 2.5. qRT-PCR

Total RNA was isolated with TRIzol reagent (Invitrogen, Carlsbad, CA, USA) according to the manufacturer’s instructions. RNA quality was assessed with the Thermo NanoDrop 2000 spectrophotometer to assess 260/280 and 260/230 nm ratios. All RNA samples met a 260/280 ratio >2.0 and 260/230 ratios in the range of 2.0–2.2. cDNA was generated from reverse transcription of 2μg total RNA using a Transcriptor First Strand cDNA Synthesis Kit (TransGen Biotech, Beijing, China). cDNA was quantified using the SYBR Green assay, and the relative gene expression levels were calculated against GAPDH or U6 by using the ∆∆Ct method. The primers we used for qRT-PCR were as follows: *IL-6* forward TACCACTTCACAAGTCGGA, *IL-6* reverse AATTGCCATTGCACAACTC; *IL-1β* forward CCTCAAAGGAAAGAATCTATACCTG, *IL-1β* reverse CTTGGGATCCACACTCTCC; *TNFα* forward TTCTCATTCCTGCTTGTGG, *TNFα* reverse TTGGGAACTTCTCATCCCT; human *STING* forward CACATCCACTCCAGGTACC, human *STING* reverse AGAAATAGATGGACAGCAGCA; mouse *STING* forward CTCATTGTCTACCAAGAACCC, mouse *STING* reverse TTCTTCCTGACGAATGTGC; let-7a forward ggcgTGAGGTAGTAGGTTGTATA, let-7b forward ggcTGAGGTAGTAGGTTGTGTG, let-7c forward ggccTGAGGTAGTAGGTTGTATG, let-7e forward ggcTGAGGTAGGAGGTTGTATA, let-7f forward ggcggTGAGGTAGTAGATTGTATA, let-7g forward ggcgTGAGGTAGTAGTTTGTACA, let-7i forward ggcTGAGGTAGTAGTTTGTGCT, common miRNA reverse GCAGGGTCCGAGGTATTC.

### 2.6. Immunohistochemistry

Mice were anesthetized and transcardially perfused with cold phosphate-buffered saline (PBS), followed by 4% paraformaldehyde (PFA) in PBS (pH 7.4). Brains were dissected out and postfixed in 4% PFA overnight. Brains were then equilibrated in 30% sucrose and sectioned into segments 40 μm-thick. Brain sections were washed three times (10 min each) with PBS and then blocked in 3% BSA (*w*/*v*), 0.3%Triton X-100, and 0.2% sodium azide for 2 h at room temperature. The primary antibodies we used were as follows: anti-Iba1 (Wako, Richmond, VA, USA; #019-19741; 1:1000), anti-GFAP (Proteintech, Rosemont, IL, USA; 16825-1-AP; 1:1000), anti-NeuN (Millipore, Hongkong, China; ABN78; 1:1000). After overnight incubation at 4 °C with primary antibodies and washing with PBS for 30 min, brain sections were incubated with the secondary antibodies conjugated with Alexa Fluor 488 or 594 (1:500). Finally, sections were stained with DAPI and mounted on glass slides with adhesion anti-fade medium. Confocal images were obtained on a ZEISS 710 confocal laser-scanning microscope. Image analyses and quantification were performed using ImageJ software V1.53 (NIH, Bethesda, MD, USA).

Microglia morphology was quantified as described previously [18]. Eight-bit 30 µm z-stack images of Iba1^+^ cells were acquired with no more than a 2 µm interval between planes. Images were converted to binary; soma size and branch numbers were measured using ImageJ with the plugin AnalyzeSkeleton. The glial scar size was defined as the intensely stained GFAP region [19]. The area of the glial scars was also measured using ImageJ software.

### 2.7. TUNEL Assay

Terminal deoxynucleotidyl transferase dUTP nick end labeling (TUNEL) staining (Beyotime Biotechnology, Shanghai, China) was performed to detect the neuronal apoptosis. Briefly, hippocampal tissue sections were washed for 10 min with PBS and incubated in 2% BSA and 0.25% triton X-100 for 30 min at RT. Sections were then incubated with 200 μL TUNEL reaction mixture at 37 °C for 1 h, followed by 3 washes with PBS. Subsequently, sections were incubated with anti-NeuN (Milipore, Hongkong, China; ABN78; 1:1000) antibody overnight at 4 °C. Finally, sections were washed for 10 min with PBS 3 times and then incubated with the secondary antibodies conjugated to Alexa Fluor 488 (Invitrogen, Carlsbad, CA, USA; 1:1000) at RT.

### 2.8. Western Blot

Brain tissues were lysed with RIPA buffer (P0013B; Beyotime, Shanghai, China). Protein samples were separated on 8–12% SDS-PAGE gels and transferred to polyvinylidene fluoride (PVDF) membranes (Millipore, Hongkong, China). The PVDF membranes were then blocked in TBS-T containing 3% milk and incubated with primary STING antibodies (Cell signaling, # 13647S; 1:1000) at 4 °C overnight. PVDF membranes were then incubated with horseradish peroxidase (HRP)-conjugated secondary antibodies (1:5000) at room temperature for 2 h. Finally, the immunoreactive proteins were treated with enhanced chemiluminescence reagent (Pierce ECL; Thermo Fisher, Shanghai, China). The 5200CE Tanon™ Chemi-Image System was used to obtain the images of the blots, and the band intensity of the blots was analyzed using the software ImageJ.

### 2.9. Dual Luciferase Assays

The sequences (~400 bp) incorporating the putative let-7i binding sites of the mouse and human *STING* 3′UTRs were amplified from genomic DNA by PCR and cloned into the dual luciferase reporter vector pmirGLO (Promega, Madison, WI, USA; E1330). Primers were used for the cloning 3′UTRs of *STING* as follows: mouse *STING* forward: CTGTGGTCTCCACGATGACTTGA, mouse *STING* reverse: CACCCAGGTCTCCAACCTTTAAA; human *STING* forward: CAGTGGTCTCCAAGCCTCTG, human *STING* reverse: ATGGAACATGACCAGGAGCCA. Mutagenesis of the putative let-7i binding sites on *STING* CDS or 3′UTR was performed using the Quick-Change II Site-directed Mutagenesis Kit (Stratagene, La Jolla, CA, USA) according to the manufacturer’s protocol. The primers for subcloning the mutated CDS or 3′UTR were as follows: mouse mutant *STING* forward: CATAatggagtGTTGGATGTTTGGCC, mouse mutant *STING* reverse: CCAACactccatTATGTCAGCAGTGTT; human mutant *STING* forward: TCACTGCCTatggagCCTCACG, human mutant *STING* reverse: TGAGGctccatAGGCAGTGATTATGA. All plasmid constructs were then verified by sequencing. Dual luciferase transfection assays were performed as previously described [20,21]. In brief, HEK293 cells in 12-well plates were transfected with agomir-let-7i, pmirGLO-3′UTR, mutated pmirGLO-3′UTR, or agomir control (scramble) using Lipofectamine 2000 (Invitrogen, Carlsbad, CA, USA). All Luciferase readings were recorded using the Dual-Luciferase Reporter 1000 System (Promega, Madison, WI, USA) following the manufacturer’s instructions.

### 2.10. Behavioral Tests

Mice were transported to the experimental room 24 h before the behavioral assays for acclimation. All experimental instruments were cleaned using 70% concentration of ethanol before the tests and between subjects. Behavioral tests were performed during the light phase, and videos were recorded and analyzed blindly by trained personnel using the software Smart V3.0.03 (Panlab, Barcelona, Spain).

*Rotarod test.* The accelerated rotarod task (YLS-4C, Beijing, China) is a sensitive index for evaluating cerebellar function as well as motor skill learning of the hippocampus [22,23,24]. Rotarod performances were assessed at day 15 post-injury (dpi). Every animal was placed on the spindle and allowed to remain stationary for 10 s at 0 rpm. The rotational speed was then slowly accelerated from 0 to 40 rpm in 300 s. The mouse remained on the device at the maximum speed until the 5-min test period elapsed. Each mouse received four trials with 30 min between trials.

*Barnes maze test.* The Barnes maze test was performed at 17–21 dpi in a 120-cm diameter circular platform that had 20 evenly-spaced holes (2 cm away from the edge, 5 cm in diameter) as described previously [21,25]. Briefly, on the habituation day (17 dpi), mice were placed in the center underneath a clear glass beaker for 30 s while white noise was played. Then, the mice were guided slowly by moving the glass beaker to the target hole that leads to the hiding box and allowed to stay in the hiding box for 1 min before being returned to the holding cage. In the training phase (18–19 dpi), mice were placed in the center of the Barnes maze, and the noise buzzer was turned on. Mice were allowed to explore the maze for 2 min. If a mouse found the target hole and entered the hiding box, the buzzer was turned off. If it did not enter the hiding box within 2 min, it was nudged until it did. There were 5 trials for training: 3 trials on training day 1 and 2 trials on training day 2. On the probe day (21 dpi), 48 h after the last training, the hiding box was removed, and mice were allowed to explore the maze for 2 min with white noise playing.

### 2.11. Statistical Analyses

All statistical data analyses were performed using the software GraphPad Prism v7.2 (GraphPad, San Diego, CA, USA). Datasets were analyzed for significance using a One-Way ANOVA with Dunnett’s multiple comparisons test. Samples sizes are provided in the figure legend. All data are presented as mean ± SEM. When a *p*-value is less than 0.05, the results are determined as statistically significant.

## 3. Results

### 3.1. Let-7i Is Downregulated in the Early Stages of TBI

**Figure 1:**
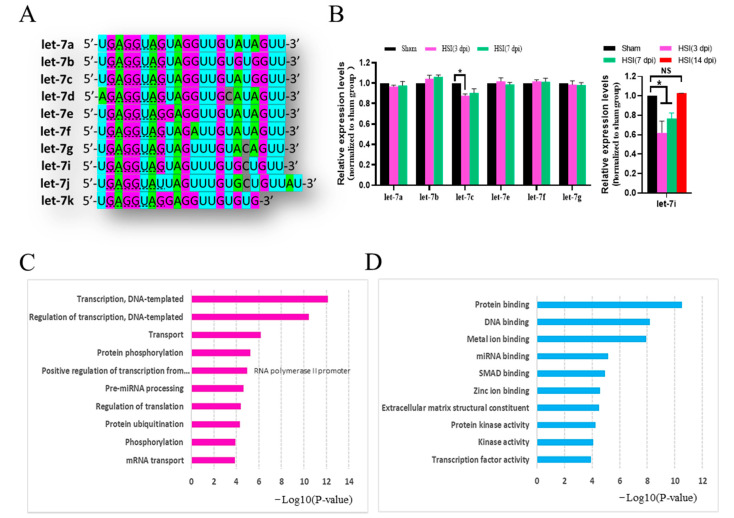
Let-7i was significantly downregulated in the hippocampus at days 3 and 7 post-injury. (**A**) Let-7 family members share the same “seed sequence” (dashed underline) and are slightly distinct from each other by a few ribose nucleotides. (**B**) Relative expression levels of let-7 family members in the hippocampus were detected by qRT-PCR. U6 was used as the internal control. All data are presented as the mean ± SEM. n = 4 mice per group. NS, non-significant; * *p* < 0.05. (**C**,**D**) Top ten biological processes (**C**) and molecular functions (**D**) of let-7i predicted targets annotated by the Gene-ontology (GO) enrichment analysis.

Let-7 is a family of miRNAs that contain a similar “seed sequence” that spans from nucleotide 2 to 8 in mammals (Figure 1A) [10]. Although mature let-7 miRNAs are encoded by different genomic loci, the conserved “seed sequence” feature suggests that the let-7 family members may have similar target mRNAs and functions [26].

To address the role of let-7 in the pathogenesis of TBI, we examined mature let-7 miRNAs in the brain after hippocampal stab injury (HSI) by using qRT-PCR. The expression of let-7i at 3 and 7 dpi was significantly lower than that in the sham group, and recovered to the same level as that in the sham group at 14 dpi (*F*_(3,8)_ = 101.2, *p* < 0.01; Sham vs. HSI(3 dpi), *p* < 0.01; Sham vs. HSI(7 dpi), *p* < 0.01; Sham vs. HSI(14 dpi), *p* = 0.986) (Figure 1B). Furthermore, let-7c expression was only downregulated at 3 dpi, while the expressions of other let-7 miRNAs were not significantly altered at either 3 or 7 dpi.

There are 1076 transcripts with conserved sites that are predicted targets of let-7i by TargetScan Release 7.2 (http://www.targetscan.org, accessed on 1 October 2020). Gene Ontology (GO) term analyses (http://geneontology.org/, accessed on 1 October 2020) found that these predicted targets were related to biological functions involving transcription, regulation of transcription, transport, protein phosphorylation, pre-miRNA processing, protein ubiquitination, phosphorylation, and mRNA transport (Figure 1C), and to molecular functions involving protein binding, DNA binding, metal ion binding, miRNA binding, SMAD binding, zinc ion binding, extracellular matrix structural constituent, protein kinase activity, kinase activity, and transcription factor activity (Figure 1D), strongly suggesting that let-7i plays an important role in the pathogenesis of TBI and may be a possible target for treating TBI.

### 3.2. Let-7i Can Be Efficiently Delivered to the Brain by Intranasal Administration of Agomir

**Figure 2:**
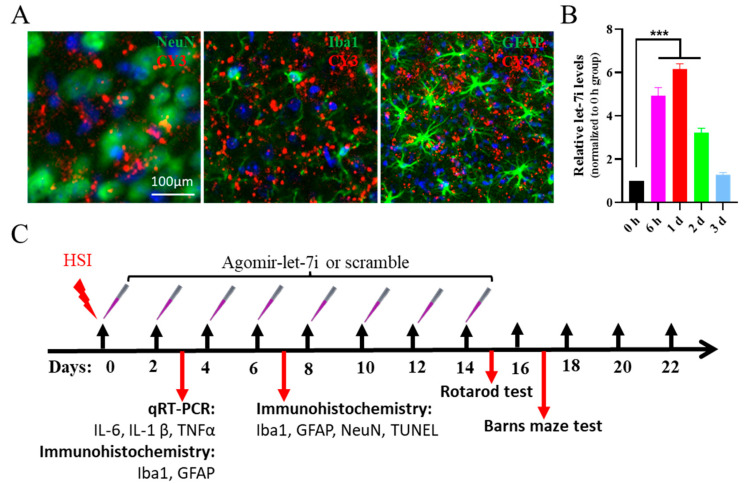
Effective delivery of agomir-let-7i to the cortex via the nasal route and timeline of the HSI experiment. (**A**) Cortex of CY3-labeled agomir-let-7i treatment of C57BL/6 mice at day 1 after nasal drug delivery. Nuclei are stained blue, and CY3-labeled agomir-let-7i displays red fluorescence. (**B**) Expression levels of mature let-7i in the cortex of C57BL/6 mice with a single dose at different time points detected by qRT-PCR analysis. All data are presented as the mean ± SEM. n = 4 mice per group. *** *p* < 0.001. (**C**) Timeline of the HSI experiment. Two-month-old male mice were assigned to undergo TBI or sham surgery. Agomir-let-7i or scramble was delivered to the brain every other day for a total of 14 days. qPCR analysis of pro-inflammatory mediators (IL-6, IL-1β, and TNF-α) and immunohistochemistry of microglia (Iba1^+^) and astrocytes (GFAP^+^) were performed at day 3 after HSI (n = 4 mice per group). Neuronal apoptosis (TUNEL^+^NeuN^+^) and glial scar sizes were measured at day 7 after HSI (n = 4 mice per group). The remaining mice were tested on the rotarod and in the Barnes maze at days 15–16 and 17–21 after HSI, respectively (n = 8 mice per group).

Next, we aimed to assess whether the upregulation of let-7i is beneficial for inhibiting the progression of TBI. A let-7i agomir was used to evaluate its therapeutic potential in the HSI mouse model by a nasal-to-brain pathway which has been proven as an effective drug delivery route to the brain [27]. As shown in Figure 2, after the intranasal administration of 1 nmol CY3-labeled agomir-let-7i, red fluorescence could be detected in NeuN^+^ neurons, GFAP^+^ astrocytes, and Iba1^+^ microglia (Figure 2A). qRT-PCR analysis showed that the expression level of mature let-7i reached a 4.93-fold peak at 6 h and a still 3.23-fold peak in the hippocampi at 2 days compared with that of the scramble control (*F*_(4,10)_ = 101.2, *p* < 0.001; 0 h vs. 6h, *p* < 0.001; 0 h vs. 1 d, *p* < 0.001; 0 h vs. 2 d, *p* < 0.001; 0 h vs. 3 d, *p* = 0.790) (Figure 2B). These data suggest that agomir-let-7i could be effectively delivered to the brain via the nasal route.

### 3.3. Agomir-let-7i Suppresses HSI-Induced Neuroinflammation and Glial Scar Formation

**Figure 3:**
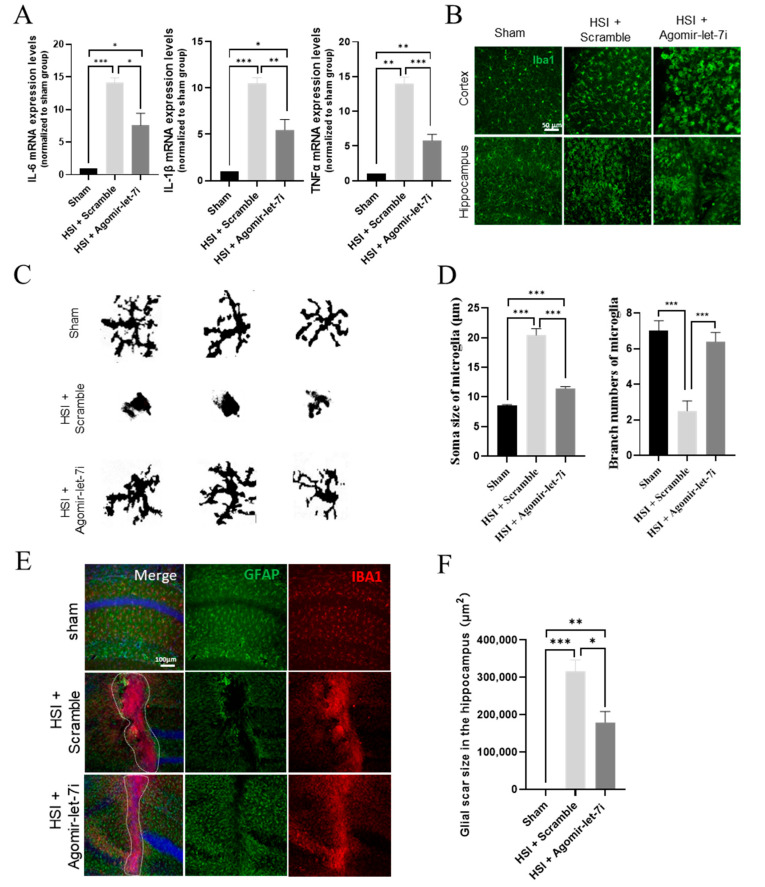
Agomir-let-7i suppressed HSI-induced neuroinflammation. (**A**) qPCR analysis showing the relative mRNA levels of the pro-inflammatory mediators IL-6, IL-1β, and TNF-α in the hippocampi of agomir-let-7i-treated and scramble control-treated mice at day 3 after HSI. Data are expressed as mean ± SEM. GAPDH was used as the internal control. (**B**) Representative images of Iba1+ microglia in the impacted and peri-lesional areas at day 3 after HSI. Scale bar, 50 µm. (**C**) The binary transformation of the microglia morphology. (**D**) Quantification of the soma size and branch numbers of microglia at the lesion site. (**E**) Representative images of GFAP and Iba1 immunohistochemistry staining of the hippocampi at day 7 after HSI. Scale bar, 100µm. (**F**) Quantification of the glial scar size in the hippocampi at day 7 after HSI. The dotted lines indicate the boundary of glial scars. Data are presented as mean ± SEM. n = 3 mice per group. * *p* < 0.05, ** *p* < 0.01, *** *p* < 0.001.

A robust inflammatory response often develops acutely post-TBI and is known to be a key secondary injury factor that can drive ongoing neuronal injury [28,29]. To determine the effects of agomir-let-7i on neuroinflammation, we performed qRT-PCR analysis to examine the expression levels of *TNF-α*, *IL-1β*, and *IL-6* in the hippocampi of agomir-let-7i-treated and scramble-treated mice at day 3 after HSI. Consistent with our expectation, the levels of *TNF-α*, *IL-1β*, and *IL-6* were significantly elevated in the hippocampi of negative control mice after HSI (Figure 3A). However, agomir-let-7i treatment exhibited dramatically lower levels of pro-inflammatory chemokines *IL-6* (*F*_(2,6)_ = 32.44, *p* < 0.001; Sham vs. HSI + Scramble, *p* < 0.001; Sham vs. HSI + Agomir-let-7i, *p* < 0.05; HSI + Scramble vs. HSI + Agomir-let-7i, *p* < 0.05), *IL-1β* (*F*_(2,6)_ = 41.83, *p* < 0.001; Sham vs. HSI + Scramble, *p* < 0.001; Sham vs. HSI + Agomir-let-7i, *p* < 0.05; HSI + Scramble vs. HSI + Agomir-let-7i, *p* < 0.01), and *TNFα* (*F*_(2,6)_ = 79.65, *p* < 0.001; Sham vs. HSI + Scramble, *p* < 0.001; Sham vs. HSI + Agomir-let-7i, *p* < 0.01; HSI + Scramble vs. HSI + Agomir-let-7i, *p* < 0.001) after HSI (Figure 3A), suggesting that agomir-let-7i has an anti-inflammatory effect in response to brain injury.

Next, we performed immunohistochemistry staining of hippocampal tissues to determine whether agomir-let-7i treatment affects microglial morphology. As shown in Figure 3B, microglia in the sham group exhibited ramified morphology with smaller soma and more microglial processes, while microglia in HSI groups displayed an amoeboid morphology, with retracted, thickened processes and enlarged soma (Figure 3B–D). Compared to the scramble-treated HSI group, the microglial soma size was significantly smaller (*F*_(2,526)_ = 42.42, *p* < 0.001; Sham vs. HSI + Scramble, *p* < 0.001; Sham vs. HSI + Agomir-let-7i, *p* = 0.195; HSI + Scramble vs. HSI + Agomir-let-7i, *p* < 0.001) and microglial branch number was greatly increased (*F*_(2,14)_ = 32.44, *p* < 0.001; Sham vs. HSI + Scramble, *p* < 0.001; Sham vs. HSI + Agomir-let-7i, *p* = 0.741; HSI + Scramble vs. HSI + Agomir-let-7i, *p* < 0.001) in the agomir-let-7i-treated HSI group (Figure 3B–D), further proving that agomir-let-7i has an anti-inflammatory effect against brain damage.

Immunohistochemistry staining analysis of GFAP (an astrocyte marker) and Iba1 (a microglia marker) revealed a considerable reduction in the size of the glial scar after agomir-let-7i treatment in comparison to the scramble control, while the sham group did not reveal any glial scar formation in the hippocampus (*F*_(2,6)_ = 43.44, *p* < 0.001; Sham vs. HSI + Scramble, *p* < 0.001; Sham vs. HSI + Agomir-let-7i, *p* < 0.01; HSI + Scramble vs. HSI + Agomir-let-7i, *p* < 0.05) (Figure 3E,F). These data indicate that the intranasal administration of agomir-let-7i inhibited the formation of glial scars after HSI.

### 3.4. Agomir-let-7i Reduces Neuronal Apoptosis after HSI

**Figure 4:**
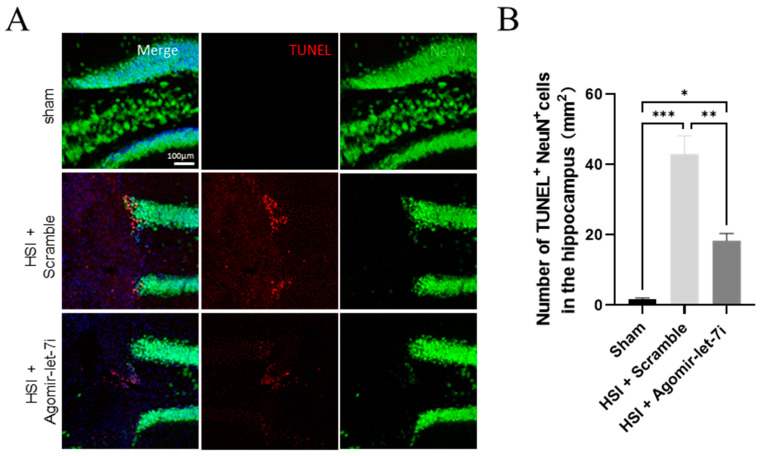
Agomir-let-7i reduced neuronal apoptosis after HSI. (**A**,**B**) Representative images (**A**) and quantification of TUNEL (red) staining (**B**) in the cortex at day 7 post-HSI. Compared with scramble treatment, agomir-let-7i administration significantly reduced the number of neurons that underwent apoptosis (TUNEL^+^NeuN^+^) in response to HSI. Data are presented as mean ± SEM. n = 3 mice per group. * *p* < 0.05, ** *p* < 0.01, *** *p* < 0.001.

To evaluate the effect of agomir-let-7i on neuronal apoptosis following HSI, we conducted terminal deoxynucleotidyl transferase dUTP nick end labeling (TUNEL) staining of hippocampal tissue sections from sham, scramble-treated, and agomir-let-7i-treated mice at day 7 post-injury. The number of TUNEL^+^/NeuN^+^ cells was significantly decreased in the agomir-let-7i-treated group compared to that in the scramble-treated group (*F*_(2,6)_ = 41.55, *p* < 0.001; Sham vs. HSI + Scramble, *p* < 0.001; Sham vs. HSI + Agomir-let-7i, *p* < 0.05; HSI + Scramble vs. HSI + Agomir-let-7i, *p* < 0.01) (Figure 4A,B), indicating that the intranasal administration of agomir-let-7i reduced neuronal apoptosis after HSI.

### 3.5. Agomir-let-7i Improves Cognitive Function in HSI Mice

Given that agomir-let-7i could reduce neuronal apoptosis as well as inflammatory response and glial scar size in the hippocampus, we speculated that agomir-let-7i-treated mice might perform better than scramble-treated mice on learning and memory tests. To test this hypothesis, we conducted the rotarod test and the Barnes maze test to evaluate hippocampal integrity in HSI mice beginning at 15 dpi.

**Figure 5:**
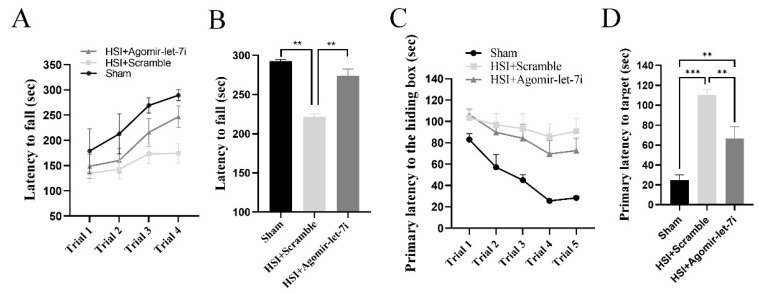
Mice with an HSI exhibit voluntary movement and forepaw grip deficits. (**A**) The averages for latency to fall from an accelerating rotarod across four rotarod test trials for sham mice, scramble-treated, and agomir-let-7i-treated HSI mice. (**B**) Agomir-let-7i-treated animals displayed less impairment on the rotarod after HSI than did the scramble-treated animals at 15 dpi. (**C**) During the training phase of the Barnes maze test, mice in every group showed improved latency of first entrance into the hiding box, but agomir-let-7i-treated HSI mice spent less time locating the hiding box compared with scramble-treated HSI mice. (**D**) In the probe test, HSI mice had a significantly longer latency to locate the hiding box (**B**) compared with their sham controls, but scramble-treated HSI mice spent a longer time reaching the target compared to agomir-let-7i-treated HSI mice. Data are presented as mean ± SEM. n = 8 mice per group. ** *p* < 0.01, *** *p* < 0.001.

In the rotarod test, although the amount of time mice spent on the rotarod increased with training in every treatment group (Figure 5A), HSI mice with agomir-let-7i treatment exhibited significantly longer latency to fall off the rotarod than scramble-treated HSI mice (*F*_(2,9)_ = 173.0, *p* < 0.001; Sham vs. HSI + Scramble, *p* < 0.001; Sham vs. HSI + Agomir-let-7i, *p* < 0.01; HSI + Scramble vs. HSI + Agomir-let-7i, *p* < 0.001) (Figure 5B), suggesting that the intranasal administration of agomir-let-7i enhanced the motor skill learning ability of HSI mice.

During the training phase of the Barnes maze test, mice in every group spent less time finding and entering the hiding box, but agomir-let-7i-treated HSI mice displayed improved first latency to locate the hiding box compared with scramble-treated HSI mice (Figure 5C). Consistently, agomir-let-7i-treated HSI mice had a significantly shorter latency to locate the hiding box in the probe test compared to scramble-treated HSI mice (*F*_(2,21)_ = 26.36, *p* < 0.001; Sham vs. HSI + Scramble, *p* < 0.01; Sham vs. HSI + Agomir-let-7i, *p* < 0.05; HSI + Scramble vs. HSI + Agomir-let-7i, *p* < 0.01) (Figure 5D). Altogether, these behavioral data illustrate that the intranasal administration of agomir-let-7i improves cognitive function in HSI mice.

### 3.6. STING Is a Direct Downstream Target of Let-7i after HSI

**Figure 6:**
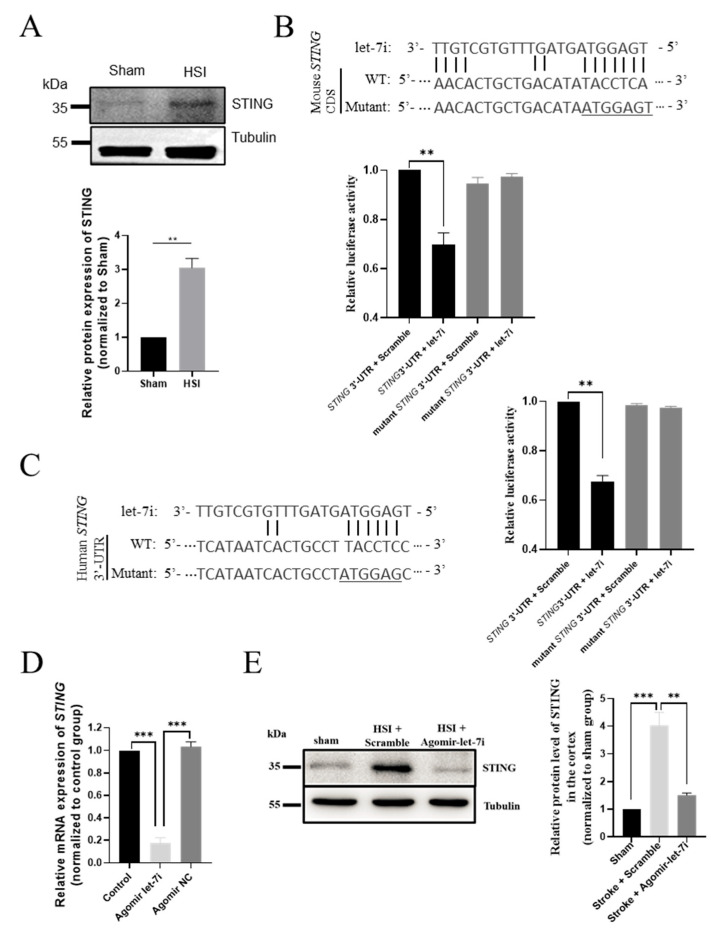
*STING* is a direct downstream target of let-7i after HSI. (**A**) Western blot analysis showing the relative protein levels of STING in sham and HSI hippocampi. Upper panel, representative images of Western blot. Lower panel, quantification data of Western blot. (**B**) Let-7i regulates the expression of mouse *STING* through the predicted binding site in the 3′UTR of *STING*. Upper panel, the let-7i binding site in the *STING* 3′UTR was mutated in the mutant plasmid. Lower panel, HEK-293 T cells were co-transfected with a luciferase reporter construct containing wild-type or mutated mouse *STING* 3′UTR and assessed for luciferase activity 72 h after transfection. (**C**) Let-7i targets human *STING* through the predicted binding site in the *STING* 3′UTR. Left panel, the let-7i binding site in the wild-type and mutated *STING* 3′UTR. Right panel, HEK-293 T cells were co-transfected with a luciferase reporter construct containing wild-type or mutated human *STING* 3′UTR and assessed for luciferase activity 72 h after transfection (n = 3). (**D**) qPCR analysis showing the relative mRNA levels of *STING* in the hippocampi of sham, scramble-treated, and agomir-let-7i-treated HSI mice. GAPDH was used as the internal control. (**E**) Western blot showing the relative protein levels of STING in the hippocampi of sham, scramble-treated, and agomir-let-7i-treated HSI mice. Tubulin was used as the internal control. Data are presented as mean ± SEM. n = 3 mice per group. ** *p* < 0.01, *** *p* < 0.001.

To further explore the molecular mechanism underlying the effect of agomir-let-7i on inflammation and neuronal apoptosis, we performed a combined computational and experimental study to identify the downstream targets of let-7i in the brain. Among the prominent candidates, *STING* (*TMEM173*) is known to play a pivotal role in responding to pathogenic DNA and self-DNA in the context of neurodegenerative and autoimmune disorders [30,31,32]. Our Western blot analysis demonstrated that the protein level of STING was significantly increased in the hippocampus after HSI (Figure 6A). Considering that let-7i was dramatically downregulated following HSI, we speculated that STING might be a key downstream target of let-7i in the injured brain. Indeed, there was a potential binding site of let-7i on the coding sequence (CDS) of mouse STING mRNA (position 343-349; Figure 6B) as well as on the 3′-UTR sequence of human *STING* mRNA (position 229-234; Figure 6C). To determine whether let-7i directly targets *STING* mRNA, we cloned the wild-type or mutated CDS of *STING* containing the predicted let-7i target site into a dual luciferase reporter construct, which allowed us to examine STING protein translation by measuring luciferase activities. We found that let-7i could significantly repress the expression of Firefly luciferase through the CDS of *STING*, while let-7i had no effect on luciferase activity when the conserved binding site was mutated (Figure 6B,C).

Next, we examined whether there are any expression changes in STING in agomir-let-7i-treated HSI brains. Consistent with our expectations, a reduced *STING* mRNA expression level was observed in the hippocampi of agomir-let-7i-treated HSI mice compared to scramble-treated HSI mice (*F*_(2,6)_ = 169.3, *p* < 0.001; Sham vs. HSI + Scramble, *p* < 0.001; Sham vs. HSI + Agomir-let-7i, *p* = 0.803; HSI + Scramble vs. HSI + Agomir-let-7i, *p* < 0.001) (Figure 6D). Moreover, Western blotting clearly showed that the STING protein level was dramatically decreased in the hippocampi of agomir-let-7i-treated HSI mice compared to scramble-treated HSI mice (*F*_(2,6)_ = 35.86, *p* < 0.001; Sham vs. HSI + Scramble, *p* < 0.001; Sham vs. HSI + Agomir-let-7i, *p* = 0.436; HSI + Scramble vs. HSI + Agomir-let-7i, *p* < 0.01) (Figure 6E). Altogether, these data support the idea that *STING* is a direct downstream target of let-7i in the injured brain.

### 3.7. Agomir-let-7i Decreased Brain Damage after PVD Stroke in Mice

**Figure 7:**
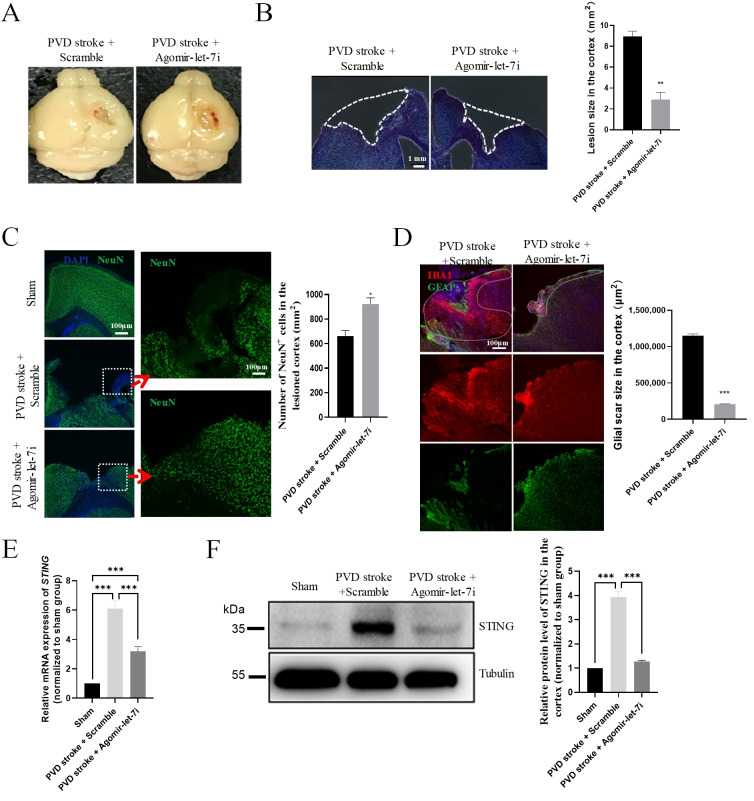
Agomir-let-7i improves recovery after a PVD stroke. (**A**,**B**) Representative images of PBS-perfused brains (**A**) and DAPI–stained sections and quantified lesion size (**B**) in brains from mice treated with agomir-let-7i and those treated with the scramble control. Lesion sizes were measured at 15 days after PVD stroke. The dotted lines indicate the boundary of lesion area. Scale bar, 1 mm. (**C**) Representative images and quantification of NeuN (green) staining in the cortex at 15 days after PVD stroke. The regions within the dotted white boxes are shown at a higher magnification (right panels). Scale bar, 100 µm. (**D**) Representative images of GFAP and Iba1 immunohistochemistry staining and quantification of the glial scar size in the cortex at 15 days after PVD stroke. The dotted lines indicate the boundary of glial scars. (**E**) qPCR analysis showing the relative mRNA levels of *STING* in the cortex of sham, scramble-treated, and agomir-let-7i-treated PVD stroke mice. GAPDH was used as the internal control. (**F**) Western blot showing the relative protein levels of STING in the cortex of sham, scramble-treated, and agomir-let-7i-treated PVD stroke mice. Tubulin was used as the internal control. Data are presented as mean ± SEM. n = 3 mice per group. * *p* < 0.05, ** *p* < 0.01, *** *p* < 0.001.

Given that agomir-let-7i treatment was neuroprotective in HSI mice, we then examined whether the intranasal administration of agomir-let-7i promotes recovery from PVD stroke. PVD stroke was induced in mice, and mice were then treated intranasally with agomir-let-7i or scramble every other day for a total of 14 days, followed by assessments of lesion size and neuronal survival. The mean lesion size measured at 15 days after PVD stroke was significantly smaller in the agomir-let-7i group compared with the scramble group (by 36.1%) (Figure 7A,B). Furthermore, the number of survived neurons was significantly increased (Figure 7C), and glial scarring was dramatically reduced (Figure 7D) in the cortex with the administration of agomir-let-7i. Again, we observed that both mRNA (*F*_(2,6)_ = 159.2, *p* < 0.001; Sham vs. PVD stroke + Scramble, *p* < 0.001; Sham vs. PVD stroke + Agomir-let-7i, *p* < 0.001; PVD stroke + Scramble vs. PVD stroke + Agomir-let-7i, *p* < 0.001) and protein levels (*F*_(2,6)_ = 360.4, *p* < 0.001; Sham vs. PVD stroke + Scramble, *p* < 0.001; Sham vs. PVD stroke + Agomir-let-7i, *p* = 0.138; PVD stroke + Scramble vs. PVD stroke + Agomir-let-7i, *p* < 0.001) of STING were significantly decreased in the cortex of agomir-let-7i-treated PVD stroke mice (Figure 7E,F). Therefore, these data suggest that the intranasal administration of agomir-let-7i could promote PVD stroke recovery.

## 4. Discussion

In the present study, we found that TBI decreased let-7i and that the intranasal administration of agomir-let-7i reduced brain damage in TBI mice as well as in PVD stroke mice. Agomir-let-7i administration improved cognitive function in brain-injured mice. Mechanistically, agomir-let-7i administration suppressed neuroinflammation, glial scar formation, and neuronal apoptosis after brain injury, suggesting that agomir-let-7i may serve as a potential therapeutic candidate against injury-induced neuroinflammatory and neurodegenerative diseases, such as TBI and PVD stroke.

Both TBI and PVD stroke have primary and secondary injury phases regardless of the severity of the insult. The primary injury encompasses mechanical damage to the brain tissues that release signals that activate microglia, astrocytes, and infiltrated peripheral immune cells to initiate the secondary injury, including inflammation, excitotoxicity, mitochondrial impairment, and neuronal cell death. The secondary injury phase evolves over minutes to days to months after the primary injury, suggesting that early interventions may ameliorate brain damage and stimulate neural regeneration and repair [33]. The main finding of this study was that the intranasal administration of agomir-let-7i could immediately increase let-7i levels, and the restoration of let-7i levels was beneficial for decreasing brain damage after TBI or PVD stroke. Great strides have been made in miRNAs as diagnostic biomarkers and therapeutic targets for TBI and stroke [34,35,36,37,38]; however, the use of miRNA for the treatment of neurotrauma and stroke is still in its nascent stage. Given that miRNA targets multiple genes at the same time, selecting those miRNAs that are highly expressed in the healthy brain but significantly downregulated following injury to examine their therapeutic potential may be more promising for the early treatment of neurotrauma because the brain may already have innate tolerance for the restoration of high expression levels of these miRNAs.

Human TBI is a sophisticated disease process, and the diversity in the extent of injury as well as in pathoanatomical subtypes means that different patients are likely to experience different courses and outcomes of TBI. In addition, it is still difficult to generate animal models that can fully recapitulate all the pathophysiological aspects of human TBI. Let-7i has been considered as a promising serum biomarker for stroke and blast-induced TBI. For example, the expression of let-7i is upregulated in blood samples collected from patients with post-stroke cognitive impairment compared with patients with post-stroke cognitive normality [39]. Similarly, let-7i is also elevated in the serum samples of rats that were exposed to three serial 120-kPa blast overpressure exposures between 3 and 24 h post-injury [11]. Surprisingly, however, we found that let-7i was downregulated in the hippocampi at days 3 and 7 post-HSI. Given that HSI causes a severe lesion of the hippocampus as well as the adjacent cortex, the reasons for the discrepancy of let-7i expression levels between other studies and ours may be multiple, such as different types of brain injury and/or the variety of tissues sampled for analysis. As let-7 is ubiquitously expressed in most somatic cells and the misregulation of let-7 leads to cancers [40], future investigations considering different tissues in various TBI and stroke models are required to determine the safe dosage range for let-7i before translating mouse models to human patients.

Neuroinflammation plays an essential role in the pathophysiology of TBI and stroke. Although a low degree of neuroinflammation is initially beneficial for debris clearance and repair, a high degree of neuroinflammation elicits secondary injury that leads to chronic inflammation and neurodegeneration [41]. Mechanistically, we found that the stimulator of interferon genes (*STING*) was a downstream target of let-7i in the brain, and the intranasal administration of agomir-let-7i could suppress the upregulation of STING, neuroinflammation, and glial scar formation in both TBI and PVD stroke mice. These findings are consistent with previous work that identified STING as a key regulator of inflammation in both rodents and human brain organoids and found that the inactivation of STING attenuates inflammation [31,32,42,43]. Importantly, STING expression is elevated in postmortem human TBI brains [43], and several studies suggest that STING deletion improves recovery outcomes after brain injury. STING deletion reduces lesion volume and polarizes microglia toward an anti-inflammatory phenotype through suppressed type I IFNs in mouse models [43]. Moreover, histone deacetylase 3 (HDAC3) activates the cGAS-STING pathway and the deletion of cGAS or HDAC3 in microglia attenuates cerebral ischemia/reperfusion-induced neuroinflammation and brain injury [44]. Although several STING antagonists (C-176, C-178, H-151, Astin C, compound 18, NO2-FAs, and SN-011) have been identified, they likely have low affinity, inactivity against human STING, and lack of specificity that limit their potential for therapeutic applications [45]. Our data strongly support that ago-let-7i can effectively inhibit both mouse and human STINGs. Given that STING inhibition is beneficial for inflammation resolution and neuronal survival, we speculate that ago-let-7i may be a promising small-molecule drug for STING-driven diseases, such as TBI and PVD stroke.

In summary, we found that brain-enriched miRNA let-7i was significantly downregulated at the early stages of TBI in mouse brains. STING was a direct downstream target of let-7i, and agomir-let-7i could protect brain tissue from neuroinflammation, glial scar formation, and neural cell death after brain injury. Overall, our data suggest that the intranasal administration of agomir-let-7i is a potential therapeutic strategy for neurotrauma and PVD stroke.

## Data Availability

The datasets used and/or analyzed during the current study are available from the corresponding author on reasonable request.
