# Correction: He et al. Intranasal Administration of Agomir-let-7i Improves Cognitive Function in Mice with Traumatic Brain Injury. *Cells* 2022, *11,* 1348

**DOI:** 10.3390/cells11203253

**Published:** 2022-10-17

**Authors:** Xuan-Cheng He, Jian Wang, Hong-Zhen Du, Chang-Mei Liu, Zhao-Qian Teng

**Affiliations:** 1 State Key Laboratory of Stem Cell and Reproductive Biology, Institute of Zoology, Chinese Academy of Sciences, Beijing 100101, China; 2 Institute for Stem Cell and Regeneration, Chinese Academy of Sciences, Beijing 100101, China; 3 Beijing Institute for Stem Cell and Regenerative Medicine, Beijing 100101, China; 4 Savaid Medical School, University of Chinese Academy of Sciences, Beijing 100049, China

The authors wish to make the following changes to their paper [1].

## 1. Text Correction

In the text of this article initially published (PDF version), there were typing errors in the last paragraph of Page 10. The text originally reading ‘Next, we examined whether there are any expression changes in STING in agomir-let-7i-treated HSI brains. Consistent with our expectations, a reduced STING mRNA expression level was observed in the hippocampi of agomir-let-7i-treated HSI mice compared to scramble-treated HSI mice (*F_(2,6)_* = 169.3, *p* < 0.001; Sham vs. HSI + Scramble, *p* < 0.001; Sham vs. HSI + Agomir-let-7i, *p* = 0.803; HSI + Scramble vs. HSI + Agomir-let-7i, *p* < 0.001) (Figure 6D).’ has been amended to read ‘Next, we examined whether there are any expression changes in STING in agomir-let-7i-treated uninjured brains. Consistent with our expectations, a reduced STING mRNA expression level was observed in the hippocampi of agomir-let-7i-treated mice compared to scramble-treated mice (*F_(2,6)_* = 169.3, *p* < 0.001; Vehicle vs. Agomir-let-7i, *p* < 0.001; Vehicle vs. Scramble, *p* = 0.803; Scramble vs. Agomir-let-7i, *p* < 0.001) (Figure 6D)’.

## 2. Error in Figure

Figures 3, 4 and 6 should be replaced. In the original publication, there were labeling errors in Figures 3 and 6. In Figure 3B, the labels ‘HIS + Scramble’ and ‘HIS + Agomir-let-7i’ were inadvertently swapped. The corrected Figure 3 appears below.

**Figure 3:**
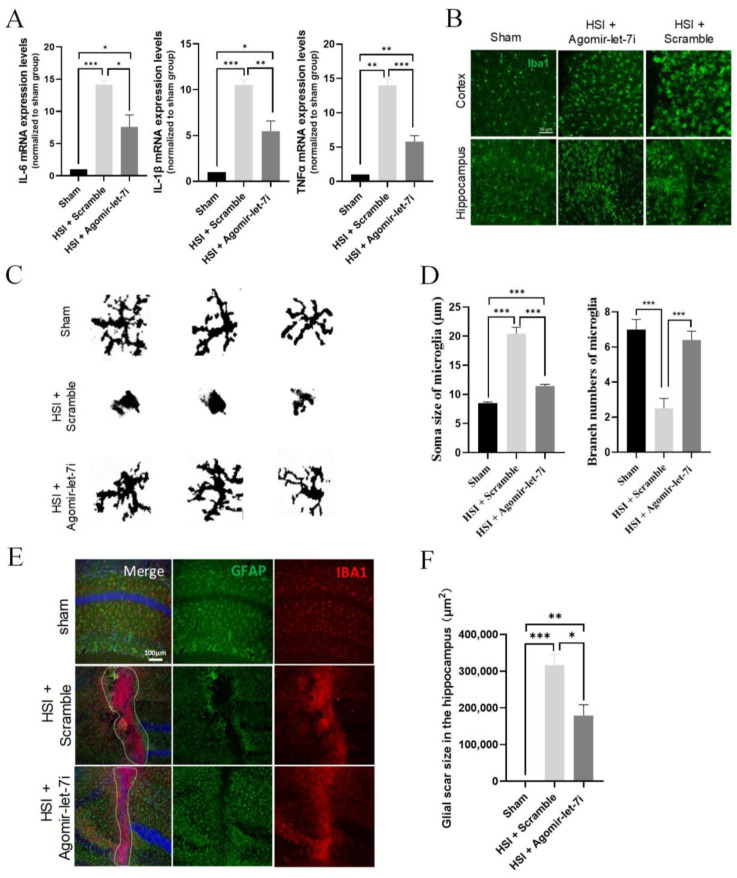
Agomir-let-7i suppressed HSI-induced neuroinflammation. (**A**) qPCR analysis showing the relative mRNA levels of the pro-inflammatory mediators IL-6, IL-1β, and TNF-α in the hippocampi of agomir-let-7i-treated and scramble control-treated mice at day 3 after HSI. GAPDH was used as the internal control. (**B**) Representative images of Iba1+ microglia in the impacted and peri-lesional areas at day 3 after HSI. Scale bar, 50 µm. (**C**) The binary transformation of the microglia morphology. (**D**) Quantification of the soma size and branch numbers of microglia at the lesion site. (**E**) Representative images of GFAP and Iba1 immunohistochemistry staining of the hippocampi at day 7 after HSI. Scale bar, 100 µm. (**F**) Quantification of the glial scar size in the hippocampi at day 7 after HSI. The dotted lines indicate the boundary of glial scars. Data are presented as mean ± SEM. *n* = 3 mice per group. * *p* < 0.05, ** *p* < 0.01, *** *p* < 0.001.

In Figure 4A of the initially published article, the separate image of TUNEL staining in the HIS + Agomir-let-7i group was erroneously flipped horizontally. The error does not affect the conclusions reported in the paper. The corrected Figure 4 appears below.

**Figure 4:**
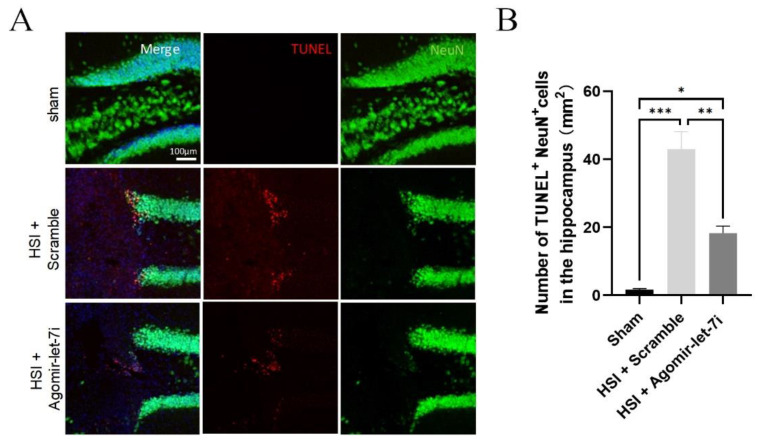
Agomir-let-7i reduced neuronal apoptosis after HSI. (**A**,**B**) Representative images (**A**) and quantification of TUNEL (red) staining (**B**) in the cortex at day 7 post-HSI. Compared with scramble treatment, agomir-let-7i administration significantly reduced the number of neurons that underwent apoptosis (TUNEL^+^NeuN^+^) in response to HSI. Data are presented as mean ± SEM. *n* = 3 mice per group. * *p* < 0.05, ** *p* < 0.01, *** *p* < 0.001.

In Figure 6D, the *x*-axis label, now reading ‘Vehicle’ and ‘Scramble’, initially appeared as ‘‘Control’ and ‘Agomir-NC’. In Figure 6E, the *x*-axis label, now reading ‘HIS + Scramble’ and ‘HIS + Agomir-let-7i’, initially appeared as ‘Stroke + Scramble’ and ‘Stroke + Agomir-let-7i’. In the Figure 6D caption, the text originally reading ‘the hippocampi of sham, scramble-treated and agomir-let-7i-treated HSI mice’ has been amended to read ‘the hippocampi of Vehicle-, scramble- and agomir-let-7i-treated uninjured mice’. The corrected Figure 6 appears below.

**Figure 6:**
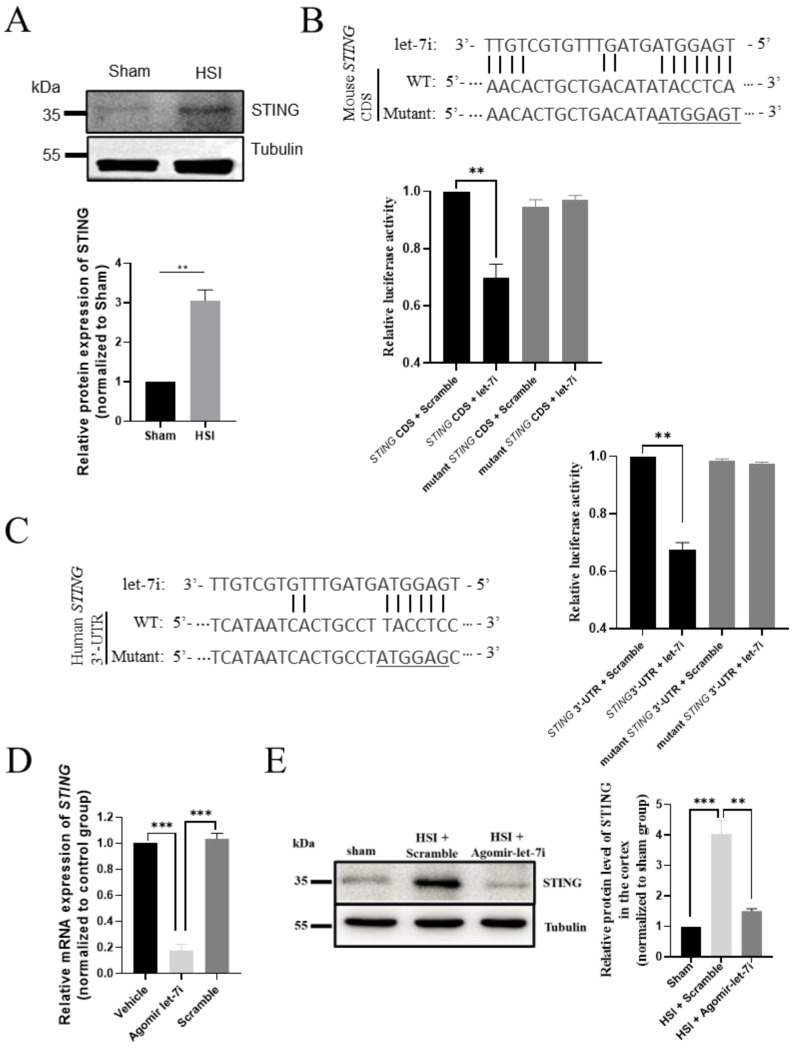
*STING* is a direct downstream target of let-7i after HSI. (**A**) Western blot analysis showing the relative protein levels of STING in sham and HSI hippocampi. Upper panel, representative images of Western blot. Lower panel, quantification data of Western blot. (**B**) Let-7i regulates the expression of mouse *STING* through the predicted binding site in the CDS of *STING*. Upper panel, the let-7i binding site in the *STING* CDS was mutated in the mutant plasmid. Lower panel, HEK-293 T cells were co-transfected with a luciferase reporter construct containing wild-type or mutated mouse *STING* CDS and assessed for luciferase activity 72 h after transfection. (**C**) Let-7i targets human *STING* through the predicted binding site in the *STING* 3′UTR. Left panel, the let-7i binding site in the wild-type and mutated *STING* 3′UTR. Right panel, HEK-293 T cells were co-transfected with a luciferase reporter construct containing wild-type or mutated human *STING* 3′UTR and assessed for luciferase activity 72 h after transfection (*n* = 3). (**D**) qPCR analysis showing the relative mRNA levels of *STING* in the hippocampi of vehicle-, scramble-, and agomir-let-7i-treated uninjured mice. GAPDH was used as the internal control. (**E**) Western blot showing the relative protein levels of STING in the hippocampi of sham, scramble-treated, and agomir-let-7i-treated HSI mice. Tubulin was used as the internal control. Data are presented as mean ± SEM. *n* = 3 mice per group. ** *p* < 0.01, *** *p* < 0.001.

The authors apologize for any inconvenience caused and state that the scientific conclusions are unaffected. This correction was approved by the Academic Editor. The original publication has also been updated.
